# Changes in Dentists' Prescribing Patterns in Norway 2005-2015

**DOI:** 10.1016/j.identj.2021.10.003

**Published:** 2021-12-03

**Authors:** Reidun Lisbet Skeide Kjome, Joachim André Johansen Bjønnes, Henning Lygre

**Affiliations:** a Centre for Pharmacy/Department of Global Public Health and Primary care, University of Bergen, Bergen, Norway; b Centre for Pharmacy/Department of Clinical Dentistry, University of Bergen, Bergen, Norway; c Department of Clinical Dentistry, University of Bergen, Bergen, Norway

**Keywords:** Dentists, Drug prescriptions, Pharmacoepidemiology, NorPD, Antibiotics

## Abstract

**Background:**

There is scant knowledge of dentists' total prescribing patterns, and little is published on this internationally. The Norwegian Prescription Database (NorPD) includes data on all dispensed prescription medication in Norway from 2004 and can be used to investigate how dentists' prescribing has changed over time. There are few Norwegian guidelines supporting dentists' prescribing, and Norwegian legislation on dentists' prescribing rights leaves room for interpretation. The aim of this study was therefore to give an overview of all prescribing from dentists in Norway in the period 2005 to 2015 and to identify trends in their prescribing pattern over this time span. We also give characteristics of the prescribing dentists.

**Methods:**

The study had a retrospective pharmacoepidemiologic design. Data on all medication prescribed by dentists and dispensed from Norwegian pharmacies in the time period 2005 to 2015 were extracted from the NorPD. Changes over time in the prescribers, patients, and medications are reported.

**Results:**

There was an increase of 50% in total number of prescriptions from dentists in Norway from 2005 to 2015; adjusted for the growth in population, there was a 33% increase. The majority of prescriptions from dentists were for antibiotics and analgesics; however, the data reveal that the dentists prescribed from all major therapeutic groups. Dentists increased antibiotic prescribing in a period when total antibiotic prescribing in Norway decreased.

**Conclusions:**

Our study finds antibiotics and analgesics dominate prescriptions from Norwegian dentists and shows an increase in use over time. It highlights the need for creating evidence-based prescribing guidelines for dentists and for ensuring that existing guidelines are implemented.

## Introduction

History suggests that drug prescription has been part of dentistry in Norway since around the year 1800.^1^ Still, there is rarely published research that describes prescription patterns amongst dental providers. Studies are generally limited to antibiotic and analgesic/anxiolytic prescribing.^2^, ^3^, ^4^, ^5^ Norwegian legislative documents regarding prescription in dentistry mandate that dentists may prescribe “drugs essential to practice dentistry,” but few specific prescribing guidelines exist. Thus, the issue of what the dentist can or cannot prescribe is debatable and, accordingly, may pose challenges for the dispensing pharmacists.

The prescribing patterns of dentists are of increasing interest as drug use in the general population is increasing. A report from The Norwegian Institute of Public Health, based on The Norwegian Prescription Database (NorPD) reveals that 70.1% of the Norwegian population had at least one drug prescription in 2018. Amongst patients >70 years, more than 90% received a drug prescription.^6^ These data expose an increased risk of dental prescriptions leading to drug–drug interactions and patients experiencing adverse reactions.

NorPD was established in 2004 and contains data, collected monthly, on all prescriptions that are dispensed from Norwegian pharmacies. Whilst many papers have been published based on data from the NorPD, few have previously aimed to describe complete prescribing patterns of Norwegian dentists,^7^ and none were found that reported changes in dentists' prescribing over time.

The aim of the present study is to describe the characteristics of prescribing dentists in Norway and how these changed over time from 2005 to 2015. Further aims are to give an overview of all prescribing from dentists in Norway in the period 2005 to 2015 and to identify trends in their prescribing pattern over this time span.

## Methods

### Study population

Our study population consisted of the following:

1) All Norwegian dentists who prescribed medication that was dispensed from a hospital or community pharmacy in the time period 2005 to 2015. Persons with dual professions (ie, as a medical doctor and dentist or veterinarian and dentist) were excluded to ensure that prescriptions were provided exclusively in a dental treatment context. Likewise, prescribers whose birth year was not available were excluded.
2) All persons who had medications prescribed by the dentists in 1), had collected the medication from a pharmacy, and had a valid ID number. NorPD does not include data from institutionalised patients in nursing homes or hospitals.

### Data

Data on all medications prescribed by dentists, and dispensed by Norwegian pharmacies, in the period 2004 to 2015 were transferred from NorPD. If a patient received a prescription that they did not collect at a pharmacy, this prescription was not included in our data. Still, in the following the term "prescribed medication" will be used for simplicity. Data were originally collected and used in the master thesis of JAJB in December 2017 at the University of Bergen, Norway.

Because data from 2004, the first year of NorPD, were not complete, we chose to exclude data from 2004 from our analysis. The raw data contained information on prescriber (pseudomised ID number, age, gender), patient (pseudomised ID number, age, gender, county), and drug (dispensing date, Anatomical Therapeutic Chemical [ATC] code, drug name, amount, package size, strength, dispensing class [A: highly addictive substances, B: other addictive substances, C: other prescription medication, F: nonprescription medication]). Data were then aggregated at 3 different levels: prescriber, patient, and drugs.

Data on total prescribing in the population were downloaded from the freely available NorPD database.^8^ The data used in this study were anonymous, as individuals were only identified by birth year, gender, and county, and ethical approval or individual consent were therefore not required.

### Statistical analysis

Data were analysed using IBM® SPSS® statistics, version 24. Descriptive statistics for the continuous variables are expressed as the median and 10th and 90th percentiles, with the exception of age, where the mean and 95% confidence interval (CI) are used. Categorical variables are expressed as frequencies and/or percentages. Figures were generated using Microsoft Excel, version 16.0 (32 bit). Extreme outliers were excluded from analysis.

## Results

### Dentists

In the period 2005 to 2015, a total of 7670 different Norwegian dentists prescribed drugs that were collected from Norwegian pharmacies. Over the whole period, 45.4% of the dentists were women, changing from 37.4% in 2005 to 50.4% in 2015. The mean age of the prescribing dentist was 49.3 (95% CI, 48.9-49.7) years in 2005 and 46.6 (95% CI, 46.3-47.0) years in 2015. The median number of prescriptions from male dentists was 29 (10th-90th percentile: 2-124) in 2005 and 46 (10th-90th percentile: 3-147) in 2015, an increase of 58.6%. Median number of prescriptions from female dentists increased from 21 (10th-90th percentile: 2-90) in 2005 to 32 (10th-90th percentile: 4-116) in 2015, an increase of 52.4%. In total, the numbers rose from 26 (10th-90th percentile: 2-111) in 2005 to 38 (10th-90th percentile: 4-141) in 2015 (46.2% increase). The highest number of prescriptions from one dentist was 1496 in 2005, whilst it was 2882 in 2015, an increase of 92.6%.

### Patients

In the period 2005 to 2015, a total of 1,183,672 patients received prescriptions from dentists in Norway, and 53% of the patients were female. Mean age of patients increased from 45.8 (95% CI, 45.7-45.9) years in 2005 to 48.4 (95% CI, 48.3-48.5) years in 2015. Number of patients who received a prescription from a dentist per year increased from 133,763 in 2005 to 193,114 in 2015, an increase of 44.4%. Prescriptions from dentists constituted 29/1000 inhabitants in 2005 and 37/1000 in 2015, an increase of 27.6%.

### Medication

**Fig. 1:**
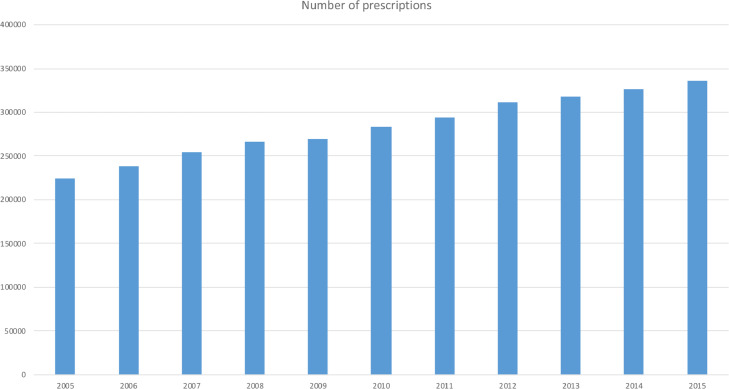
Number of prescriptions written by Norwegian dental providers over the period 2005 to 2015.

In the period 2005 through 2015, a total of 3,120,222 prescriptions were delivered from Norwegian dentists. The number of prescriptions increased from 224,501 in 2005 to 335,723 in 2015 (Figure 1). Adjusted for changes in population size, this corresponds to a change from 46.6 prescriptions/1000 inhabitants to 64.7 prescriptions/1000 inhabitants, an increase of 33.2%.

More than half (51%) of the prescriptions from dentists in the period 2005 to 2015 were in the ATC group J01 (ie, antibacterial drugs for systemic use). Throughout the period examined, phenoxymethylpenicillin (J01CE02), ibuprofen (M01AE01), and paracetamol/codeine in combination (N02AA59) were the top 3 medications prescribed by Norwegian dentists.

Phenoxymethylpenicillin (J01CE02) constituted 37.6% of all prescriptions from dentists over the period from 2005 to 2015. In 2005, 38.5% of dentists' prescriptions were for phenoxymethylpenicllin, whilst in 2015 the percentage was 33.4%. In total, the number of prescriptions in group J (anti-infectives for systemic use) from dentists increased from 110,887 in 2005 (2.4 prescriptions/1000 inhabitants) to 158,350 in 2015 (3.1 prescriptions/1000 inhabitants), an increase of 27.2% (adjusted for change in population). Table 1 shows the 6 most prescribed antibiotics in 2005 and 2015. Whilst the number of prescriptions for erythromycin and doxycyline decreased, the number of prescriptions/1000 inhabitants increased for amoxicillin by 142%, for clindamycin by 209%, and for azithromycin by 200%.

**Table 1:** The 6 most prescribed antibiotics in 2005 and 2015.

ATC code	Drug	Number of prescriptions 2005	Number of prescriptions /1000 inhabitants	Number of prescriptions 2015	Number of prescriptions /1000 inhabitants	Change in percentage, adjusted for population
J01CE02	Phenoxymethylpenicillin	86,432	18.7	112,128	21.6	15.5
J01FA01	Erythromycin	6108	1.3	4883	0.9	−30.8
J01CA04	Amoxicillin	5642	1.2	14,901	2.9	141.7
J01FF01	Clindamycin	5294	1.1	17,694	3.4	209.1
J01AA02	Doxycycline	2810	0.6	2501	0.5	−16.7
J01FA10	Azithromycin	1411	0.3	4883	0.9	200.0

Abbreviation: ATC, Anatomical Therapeutic Chemical.

In addition to prescriptions from group J, metronidazole (P01AB01) was also frequently prescribed. In 2005, a total of 8197 prescriptions for metronidazole were registered in Norway from dentists (1.8/1000 inhabitants), increasing to 12,800 (2.5/1000 inhabitants) in 2015.

From 2005 to 2015, there was an increase of 16.7% in the number of prescriptions in group M01 (anti-inflammatory and antirheumatic products) from dentists. Prescriptions for ibuprofen (M01AE01) constituted 93.5% of prescriptions in group M01 in 2005, and in 2015. Prescriptions for diclofenac (M01AB05) constituted 4.1% in 2005 and 5.3% in 2015. Table 2 shows the most commonly prescribed analgesic and anti-inflammatory drugs.

**Table 2:** All drugs from class N02 (analgesics) or class M01 (anti-inflammatory or antirheumatic products) with at least 200 prescriptions in 2005 or 2015.

ATC code	Drug	Number of prescriptions 2005	Number of prescriptions /1000 inhabitants 2005	Number of prescriptions 2015	Number of prescriptions /1000 inhabitants 2015	Change in percentage, adjusted for population
M01AE01	Ibuprofen	45,972	9.94	53,689	10.34	4.0
N02AA59	Paracetamol/codeine in combination	37,419	8.09	51,535	9.93	22.7
M01AB05	Diclofenac	2012	0.44	3061	0.59	35.5
N02BE01	Paracetamol	706	0.15	3113	0.60	292.8
M01AC01	Piroxicam	444	0.10	146	0.03	−70.7
M01AH03	Valdecoxib	294	0.06	0	0.00	−100.0
N02AX02	Tramadol	269	0.06	1383	0.27	358.0
M01AE02	Naproxen	222	0.05	223	0.04	−10.5

Abbreviation: ATC, Anatomical Therapeutic Chemical.

Whilst the majority of prescriptions from dentists were for antibiotics and analgesics, in total Norwegian dentists prescribed 776 different substances from 2005 to 2015, from all the main ATC categories (Table 3). This included prescriptions for drugs such as ethylmorphine (8247 prescriptions), chloramphenicol (2537 prescriptions), prednisolone (2105 prescriptions), metoprolol (1355 prescriptions), simvastatin (1061 prescriptions), levothyroxine sodium (594 prescriptions), estradiol (523 prescriptions), cisordinol (347 prescriptions), and sildenafil (180 prescriptions).

**Table 3:** Total number of prescriptions from dentists for the period 2005 to 2015, by main ATC group and percentage of total prescribing.

ATC group	Name	Number of prescriptions	Percentage
J	Anti-infective for systemic use	1,597,831	51.2
M	Musculoskeletal system	603,901	19.4
N	Nervous system	557,140	17.9
A	Alimentary tract and metabolism	166,524	5.3
P	Antiparasitic products, insecticides, and repellents	129,200	4.1
D	Dermatologicals	24,620	0.8
R	Respiratory system	18,399	0.6
C	Cardiovascular system	8367	0.3
S	Sensory organs	4213	0.1
B	Blood and blood forming organs	3438	0.1
H	Systemic hormonal preparations, excluding sex hormones and insulins	3167	0.1
G	Genito urinary system and sex hormones	3022	0.1
L	Antineoplastic and immunomodulating agents	262	0.0
V	Various	89	0.0
Q	Veterinary drugs	49	0.0
Total		3,120,222	100.0

Abbreviation: ATC, Anatomical Therapeutic Chemical.

#### Controlled substances (class A and B)

Only 0.1% of the prescriptions from dentists were for drugs in class A (highly addictive substances). From 2007 to 2015, there was a decreasing number of prescriptions from class A, from 301 prescriptions in 2005 to 130 prescriptions in 2015. Over the entire period, the most commonly prescribed drugs in this group were flunitrazepam (41.8% of prescriptions), morphine (12.3% of prescriptions), and oxycodone (11.6% of prescriptions).

In the period 2005 to 2015, prescriptions from class B (other addictive substances) constituted 17.2% of total prescribing from Norwegian dentists. Paracetamol/codeine in combination accounted for 91.6% of this, but dentists prescribed 21 different generic compounds from class B over this period.

**Fig. 2:**
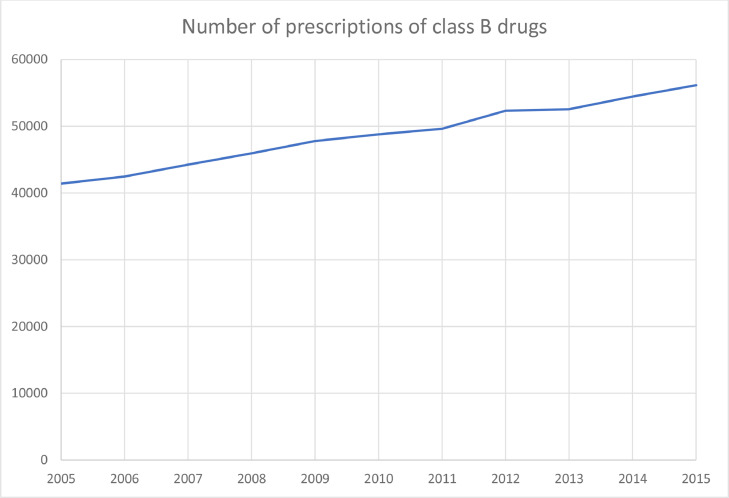
Number of dental prescriptions for class B drugs from 2005 to 2015.

From 2005 to 2015, the number of prescriptions from Norwegian dentists in class B increased from 41,397 (9.0/1000 inhabitants) to 56,136 (10.2/1000 inhabitants), corresponding to an increase of 20.8% adjusted for population (Figure 2). Codeine in combination with paracetamol (ATC code N02AA59/N02AJ06) was the dominating medication and made up 91.5% of prescribing in class B. In 2015, a total number of 367,263 persons were registered using codeine/paracetamol in Norway, and 39,895 (10.9%) of the individuals received their prescriptions from dentists. The prescribing of tramadol increased from 239 prescriptions in 2005 (0.05 prescriptions/1000 inhabitants) to 1383 prescriptions in 2015 (0.27/1000 inhabitants), an increase of 440% adjusted for the growth of the population.

Total number of prescriptions of analgesics (ie, ATC group N02, from classes A, B, and C) from dentists increased 45.5% in the period 2005 to 2015. The prescribing of drugs from group N05 (ie, psycholeptics) was quite stable over the period, with 2843 prescriptions in 2005 (covering 17 different substances) and 2305 in 2015 (covering 12 different substances). Over the entire period (2005-2015) the dentists prescribed 31 different substances within group N05.

## Discussion

We have previously reported on the total prescription from dentists in Norway 2015.^7^ Registry data have previously been used in odontology research in Norway,^9^^,^^10^ albeit to our knowledge the current paper is the first time registry data have been used to report how dentists' total prescribing changes over time.

Our report revealed an increase of over 30% in total prescriptions from dentists in Norway from 2005 to 2015. Male dentists prescribed a higher number of drugs in the whole period compared to female dentists. Antibacterial drugs and analgesics dominate prescriptions from dentists.

Whilst total Norwegian prescribing of phenoxymethylpenicillin decreased by 5% over the period,^8^ prescribing amongst dentists increased by almost 30%. We also found a large increase in the prescribing of amoxicillin and of clindamycin over the period (Table 1). This is worrying, as amoxicillin is a broad-spectrum antibiotic and not the initial drug of choice in oral infections. Clindamycin is a lincosamide and should not be the initial “drug of choice,”^11^ as it is primarily recommended for use in cases of penicillin allergies. Other countries have reported similar findings. In 2020, Smith et al. published a study comparing antibiotic prescribing in Norway and Denmark to England and Scotland, from 2010 to 2016, and found that Norwegian and Swedish dentists prescribe fewer antibiotics than UK dentists.^12^ In Germany,^13^ Halling et al. reported that 75% of antibiotic dental prescribing in the period from 2012 to 2015 consisted of clindamycin and amoxicillin. Other countries also seem to have an increased antibiotic prescription in this period. Okunseri et al.^14^ found that the amount of antibiotics prescribed at dental visits in the US increased from 2003 to 2013 and that there were racial disparities in the prescribing, with Black patients having a higher probability of being prescribed antibiotics than White patients.^14^ Though it would have been interesting to explore similar differences in the Norwegian data, the NorPD data do not contain variables that would make this possible. A current discussion worldwide on antimicrobial resistance and recent changes in antimicrobial guidelines have challenged dentists to evaluate more carefully the risk–benefit relationship of antibiotics as prophylaxis. In an online survey of Minnesota dentists, Tomczyk et al.^15^ found that dentists were reported to prescribe antibiotics for a number of reasons not supported by guidelines, such as gingival pain or legal concerns. Conflicting guidelines and conflicting scientific evidence were the most commonly stated challenges to stewardship of antibiotic use amongst the responders.^15^ However, in Norway the one clear prescribing guideline for dentists is for the prescribing of antibiotics,^11^ so this should not explain our findings. Still, it is possible that there is a lack of knowledge about the guideline amongst Norwegian dentists. Kim et al. in 2017^16^ found that one could successfully reduce antibiotic prescribing by educating dentists and distributing antibiotic prescription guidelines and also by making a simple change in the prescribing software. A reduction was achieved, especially in surgical procedures such as implant surgery and tooth extractions without infection.^16^

The second most common class of drugs prescribed by Norwegian dentists was, quite naturally, medication for pain relief, analgesics, and anti-inflammatory/antirheumatic drugs (Table 2). The use of all the drugs in Table 2 increased except valdecoxib, which was withdrawn from the market in 2005 and therefore was not prescribed in 2015, and piroxicam, for which use decreased by almost 70% from 2005 to 2015. The more than 400% increase in the prescribing of tramadol is surprising, given that it is a class B drug that dentists are not allowed to prescribe in Norway.^17^ In a study from the United States, Gupta et al.^18^ found that the number of opioid prescriptions amongst dental patients increased from 2010 to 2015, especially for children (aged 11 to 18 years). It has also been suggested by Schroeder et al.^19^ that dentists' opioid prescribing has contributed to the opioid misuse and abuse crisis in the country. Hydrocodone, the most frequently prescribed opioid in the study by Schroeder et al., is not on the market in Norway. Whilst the dentists' prescribing of class A drugs such as oxycodone was very low in our Norwegian data, it is important that dentists are aware of the addictive potential and negative side effects of all the opioids, including the so-called "weak" opioids codeine and tramadol.^20^, ^21^, ^22^

Another worrying trend is the 50% increase in the prescribing of diclofenac, as there is evidence that the initiation of diclofenac increased the risk of major adverse cardiovascular events by 50% compared to nonusers.^23^ In Norway in 2015, the Norwegian medicine information centres provided academic detailing visits to general practitioners focused on reducing potentially harmful prescribing of diclofenac.^24^ When comparing cities where general practitioners had received the visits with those who had not, and also studying changes over time, they found a significant reduction in the prescribing rates of diclofenac.^24^ In 2016 and 2017, the visits focused on the correct prescribing of antibiotics. Given the results of our study, there seems to be a need for similar services being provided to dentists. Also, a closer interprofessional collaboration between dentists and pharmacists could potentially lead to better prescribing.^25^

Norwegian legislation regarding prescription in dentistry mandates that dentists may prescribe “drugs essential to practice dentistry.” Our report proved Norwegian dentists to prescribe drugs that are clearly outside legislation. More than 8000 prescriptions in the reported period were for drugs used to treat the heart and the circulatory system (ie, ATC code C). Additionally, more than 3000 prescriptions pertaining the reproductive system were documented (ie, ATC code G). In 2018, a Norwegian dental journal published an opinion piece arguing that dentist should have expanded prescribing rights.^26^ However, other countries have chosen other approaches. In the UK, whilst registered dentists legally can prescribe from the entirety of the British National Formulary (BNF) and BNF for children,^27^ dentists prescribing within the National Health service can only prescribe drugs from the Dental Practitioners Formulary. Also, detailed guidelines support dentists in their prescribing decisions.^27^ Whilst it is outside the scope of this study to evaluate the clinical quality of individual prescribing, our findings indicate that there at least is a need for clarification of what dentists are allowed to prescribe and that Norwegian dentists may benefit from more detailed guidelines and perhaps also a clear list of which drugs they may prescribe, as is the case for medication in class A and B.

## Conclusions

Our study finds antibiotics and analgesics to dominate prescriptions from Norwegian dentists and exhibited an increase in use over time. The worldwide epidemic of opioid abuse and dependence has prompted reconsideration of existing prescribing patterns of opioid analgesics in the management of dental pain. Additionally, the global development of antibiotic resistance demands restricted use of antibacterial drugs. Our report has documented the need for creating evidence-based prescribing guidelines for dentists and for ensuring the implementation of existing guidelines.

## Conflict of interest

None disclosed.
